# Intestinal mucus-derived metabolites modulate virulence of a clade 8 enterohemorrhagic *Escherichia coli* O157:H7

**DOI:** 10.3389/fcimb.2022.975173

**Published:** 2022-08-08

**Authors:** Nicolás Garimano, María Luján Scalise, Fernando Gómez, María Marta Amaral, Cristina Ibarra

**Affiliations:** Laboratorio de Fisiopatogenia, Instituto de Fisiología y Biofísica, IFIBIO-Houssay (UBA-CONICET), Departamento de Ciencias Fisiológicas, Facultad de Medicina, Universidad de Buenos Aires, Buenos Aires, Argentina

**Keywords:** STEC, O157:H7, Shiga toxin type 2 (Stx2), intestinal mucus-derived metabolites, hemolytic uremic syndrome (HUS)

## Abstract

The human colonic mucus is mainly composed of mucins, which are highly glycosylated proteins. The normal commensal colonic microbiota has mucolytic activity and is capable of releasing the monosaccharides contained in mucins, which can then be used as carbon sources by pathogens such as Enterohemorrhagic *Escherichia coli* (EHEC). EHEC can regulate the expression of some of its virulence factors through environmental sensing of mucus-derived sugars, but its implications regarding its main virulence factor, Shiga toxin type 2 (Stx2), among others, remain unknown. In the present work, we have studied the effects of five of the most abundant mucolytic activity-derived sugars, Fucose (L-Fucose), Galactose (D-Galactose), N-Gal (N-acetyl-galactosamine), NANA (N-Acetyl-Neuraminic Acid) and NAG (N-Acetyl-D-Glucosamine) on EHEC growth, adhesion to epithelial colonic cells (HCT-8), and Stx2 production and translocation across a polarized HCT-8 monolayer. We found that bacterial growth was maximum when using NAG and NANA compared to Galactose, Fucose or N-Gal, and that EHEC adhesion was inhibited regardless of the metabolite used. On the other hand, Stx2 production was enhanced when using NAG and inhibited with the rest of the metabolites, whilst Stx2 translocation was only enhanced when using NANA, and this increase occurred only through the transcellular route. Overall, this study provides insights on the influence of the commensal microbiota on the pathogenicity of *E. coli* O157:H7, helping to identify favorable intestinal environments for the development of severe disease.

## Introduction

The human gut microbiota is a diverse and complex bacterial consortium, including beneficial commensal bacteria, mainly comprising the phyla Actinobacteria, Proteobacteria, Firmicutes and Bacteroidetes, which maintain a symbiotic relationship with their host (Rinninella et al., 2019). The normal commensal microbiota has a protective function against the pathogenic effects of bacteria such as *C. difficile*, *S. typhirmurium*, and *E. coli* through mechanisms of competition for nutrients, production of bacteriocins, inhibitory metabolic byproducts, and modulation of the host’s immune response (Abt and Pamer, 2014). There are several mechanisms used by the host to maintain the intestinal immune balance and prevent unwanted immune responses towards the commensal bacterial community, one of them being the production of mucus.

In particular the colonic mucus is made up of a polymerized and strongly O-glycosylated protein, named MUC2, which covers the entire intestinal epithelium (Johansson et al., 2011a). The colonic mucus has two layers: the inner layer, dense and firm, is in direct contact with the colonic epithelium, and is generally inaccessible to the microbiota, being sterile under normal conditions (Hansson and Johansson, 2010), and, on the other hand, the outer layer, which is less dense and is completely colonized by the commensal microbiota. A fraction of the commensal microbiota, as is the case of *B. thetaiotaomicron*, possess highly specialized mucolytic activity thanks to the production of glycosidases that release the monosaccharides present in the O- and N-glycosidic ornaments of the mucins that compose it (Sonnenburg et al., 2005; Tailford et al., 2015). The main sugars present throughout the intestinal tract that can be subsequently released from the mucolytic activity of these bacteria include N-acetylneuraminic acid (NANA), N-acetyl-glucosamine (NAG), Fucose, Galactose and N-acetylgalactosamine. (N-Gal) (Deplancke and Gaskins, 2001; Robbe et al., 2004). These sugars can then be used metabolically by the same degrading bacteria or by others that do not have this capacity, as is the case with *E. coli* (Sicard et al., 2017). Also, these can be used not only by components of the commensal microbiota, but also by pathogens such as enterohemorrhagic *E. coli* (EHEC). EHEC strains are responsible for multiple clinical syndromes, being hemolytic uremic syndrome (HUS) the most clinically significant due to its incidence and severity (Gianantonio et al., 1968; Boyce et al., 2002). *E. coli* O157:H7 is the most prevalent serotype associated with HUS and possess a chromosomal pathogenicity island, known as the locus of enterocyte effacement (LEE), which has been widely associated with severe disease (Beutin and Martin, 2012; McWilliams and Torres, 2014). The genes encoded in the LEE are responsible for intimate adhesion of EHEC to colonic epithelial cells (McWilliams and Torres, 2014), which is followed by injection of bacterial effector proteins into the host cell through a type III secretion system (T3SS) (Jerse et al., 1990). These effector proteins produce attaching and effacing (A/E) lesions on intestinal cells and interfere with host cells in many ways, inducing a profound rearrangement of cell cytoskeleton, and a loss of tight junction and membrane integrity (Ugalde-Silva et al., 2016). Additionally, EHEC can produce Shiga-toxin type 2 (Stx2), which is widely recognized as the most important virulence factor of *E. coli* O157:H7 responsible for HUS (Palermo et al., 2009). After being released in the colonic lumen, Stx2 can translocate the intestinal epithelial barrier and enter the bloodstream, causing the systemic symptoms characteristic of HUS. Several mechanisms for toxin translocation across intestinal epithelium have been proposed, either *via* the transcellular pathway, which has been described to be preceded either by internalization of Stx2 associated with its specific receptor, globotriaoscyl ceramyde (Gb3) (Müller et al., 2017), by an unspecific macropinocytic mechanism that does not involve the Gb3 receptor (Lukyanenko et al., 2011), or by the paracellular pathway, stimulated by neutrophil transmigration and actin rearrangements during EHEC infection (Hurley et al., 2001).

It has been demonstrated that EHEC can obtain a competitive advantage through the use of mucus-derived sugars, not only as a carbon source, but also as signaling metabolites of the intestinal microenvironment, being able to adapt its proliferative and virulent activity in its presence (Lee et al., 2021). In addition, EHEC preferentially uses different mucus-derived carbon sources relative to commensal *E. coli in vivo*, indicating that these sugars provide a differential niche that the pathogen exploits to increase its ability to compete with commensal microbiota, both in humans and in its bovine natural reservoir (Fabich et al., 2008; Bertin et al., 2013). EHEC regulates the expression of several of its virulence factors through environmental sensing of mucus-derived sugars. It has been shown that the presence of Fucose, NAG or NANA, metabolites derived from the mucolytic activity of *B. thetaiotaomicron* among others, repress the expression of the LEE pathogenicity island by EHEC, reducing its adhesion to the epithelium (Pacheco et al., 2012; Le Bihan et al., 2017). A deletion in any of the regulators involved in the metabolic pathways of these sugars (nagC for NAG, nanR for NANA, and fusKR for Fucose) triggers defects in EHEC colonization *in vivo*, highlighting the importance of these regulators on bacterial adaptive fitness to the intestinal environment.

It is generally accepted that this regulation, based on the sensing of sugars, results in the repression of energetically expensive virulence factors in environments where both the microbiota is present, and the epithelial surface is far from the pathogenic bacteria. When approaching the inner layer of mucus, where the concentration of these sugars is low due to a decrease in the commensal microbiota, the LEE, and other pathogenicity-related traits, such as fimbriae or biofilm formation, derepress and, thus, increase bacterial ability to adhere to the epithelium (Sohanpal et al., 2004; Kamada et al., 2012; Cameron and Sperandio, 2015; Carlson-Banning and Sperandio, 2016; Le Bihan et al., 2017; Sicard et al., 2018).

Considering the reported importance of mucus-derived sugars on EHEC colonization and pathogenicity, in this study we aim to explore the influence of the five most relevant metabolites at the intestinal level (Fucose, Galactose, N-Gal, NANA and NAG) on the production and intestinal translocation of Stx2. Likewise, we seek to evaluate whether two of these poorly explored metabolites, such as Galactose and N-Gal, influence the adhesion profile of EHEC on epithelial cells. These results will allow us to investigate the metabolic pathways most involved in the pathogenicity of EHEC and their association with the commensal microbiota present.

## Materials and methods

### Materials

Purified Stx2a was provided from Phoenix Laboratory, Tufts Medical Center, Boston, MA, USA). Five carbohydrates derived from mucus degradation found in humans were used in the assays (Robbe et al., 2004; Johansson et al., 2011a; Sicard et al., 2017). Fucose (L-Fucose), Galactose (D-Galactose), N-Gal (N-acetyl-galactosamine), NANA (N-Acetyl-Neuraminic Acid), NAG (N-Acetyl-D-Glucosamine) and an unrelated monosaccharide (Glucose, D-Glucose). All sugars were purchased from Sigma Aldrich, USA and used as carbon source in bacterial cultures at a concentration of 10 mM. All carbohydrates can be catabolized by *E. coli* O157:H7 (Carlson-Banning and Sperandio, 2016). The concentration used was similar to that reported in bovine intestinal content (Bertin et al., 2013) and used in similar systems in previous literature (Pacheco et al., 2012; Bertin et al., 2013). A fluorescein isothiocyanate (FITC)-labelled Dextran (average molecular weight of 70 kDa, Sigma Aldrich, catalog # 46945) was used as a marker of paracellular permeability (Chattopadhyay et al., 2017)

### Bacterial strains and growth condition

*E. coli* O157:H7 strain 125/99 wild type (*E. coli* O157:H7) isolated from a patient with HUS has been previously described (Rivas et al., 2006). A mutant from the parenteral *E. coli* O157:H7 strain 125/99wt lacking the *stx2* gene (*E. coli* O157:H7Δstx2) was previously obtained and described (Albanese et al., 2015). Bacterial strains were grown in Luria Broth medium at 37°C for 18 h with shaking at 150 rpm and then diluted 1/50 in minimal M9 medium (Sigma-Aldrich, USA supplemented with 2 mM MgSO_4_, 0.1 mM CaCl_2_ and 10 mM mucus-derived metabolites (Fucose, Galactose, N-Gal. NANA or NAG) or Glucose and cultured at 37°C for 5 h with shaking at 200 rpm or, in selected experiments, co-cultured for 5 h in static conditions with HCT-8 cells in M9 medium supplemented with 10.mM mucus-derived metabolites after washing with PBS to remove HCT-8 culture medium traces. Viability of HCT-8 cells co-cultured under these conditions did not show significant differences compared to growth-arrested control cells (p>0.05, NS, data not shown). Bacterial density (CFU/ml) at the end of the incubation period was estimated from a standard optical density curve of bacterial cultures in M9 medium + 10 mM Glucose. (Data not shown). *E. coli* O157:H7(SN) supernatant was collected after centrifugation at 10,000g for 5 min, followed by filtration through a 0.22 μm filter (Millipore, Billerica, MA, USA).

### Cell lines

The human intestinal cell line HCT-8 (ATCC CCL-244, Manassas, VA, USA) was maintained in RPMI-1640 medium (ATCC) and the monkey kidney cell line Vero (ATCC CCL-81) was grown in DMEM/F12 (Sigma Aldrich, St. Louis, MO USA). Both media were supplemented with 10% fetal bovine serum (FBS, Internegocios S.A., Buenos Aires, Argentina), 100 U/ml penicillin and 100 μg/ml streptomycin. Additionally, 1mM L-glutamine, 10 mM sodium pyruvate, 10 mM HEPES, 10 mM glucose were also added to HCT-8 cultures. Cells were grown at 37°C in a humidified 5% CO_2_ incubator. Cells were subcultured until 80% confluence was obtained (7-10 days) in antibiotic-free medium. For growth-arrested conditions, medium without FBS was used.

### Neutral red uptake assays and Stx2 concentration determination

Vero cells grown to subconfluence on 96-well plates were treated for 72 h under growth-arrested conditions with serial dilutions of filtered supernatants, translocated medium or commercial Stx2 of known concentration. After selected treatments, neutral red uptake cytotoxicity assay was performed as previously described, with minor modifications (Repetto et al., 2008). Cells were washed twice with PBS (145 mM NaCl, 10 mM NaH2PO_4_, pH 7.2) and incubated for 2 h with freshly diluted neutral red in PBS to a final concentration of 50 μg/ml. Cells were then washed with 1% CaCl_2_ and 4% formaldehyde twice and were then solubilized in 1% acetic acid and 50% ethanol. Absorbance at 546 nm was read in an automated plate spectrophotometer. Results were expressed as Stx2 concentration, calculated by interpolation in Stx2 standard curves.

### HCT-8 cell monolayer culture

HCT-8 were seeded on Milicell culture inserts (PIHP01250, Millipore, Billerica, MA, USA) of 12 mm diameter and 0.4 um pore size (filter area: 1.13 cm) placed on a 24-well plate and grown for about 7–10 days as previously described until a continuous monolayer was achieved. The development of monolayers was monitored measuring the transepithelial electrical resistance (TEER) with a Millicell-ERS electric resistance system (Millipore, Billerica, MA, USA) until TEER values were stable for 2 consecutive days and higher than 1,200 Ω.cm2, which is consistent with cell polarization (Hurley et al., 1999).

### Translocation assays

Briefly, after HCT-8 cell monolayer formation, complete medium was removed from upper (apical) and lower (basolateral) chambers and replaced with M9 medium. Apical side of the HCT-8 cells were exposed to Stx2 (100 ng/ml) in the presence of *E. coli* O157:H7Δstx2 (1 x 10^8^ CFU/ml) and supplemented with 10 mM of mucus-derived metabolites (Fucose, Galactose, N-Gal. NANA or NAG) or Glucose at 37°C for 5 h in a humidified 5% CO_2_ incubator. Following the incubation period, the media from the lower chamber was filter-sterilized to determine Stx2 concentration by Vero cell cytotoxic assays.

### Paracellular permeability

Paracellular permeability in HCT-8 monolayers was determined by measuring FITC-Dextran passage from the apical- to basolateral side of monolayers, taking into account that Dextran (MW 70 kDa) cannot penetrate the cellular membrane under physiological conditions, being its molecular weight similar to Stx2 (Matter and Balda, 2003). FITC-Dextran was measured according to methods described previously with some modifications (Balda et al., 1996; Garimano et al., 2019). FITC-Dextran (1 mg/ml) was added on the upper (apical) chamber at the beginning of each experiment. Following the incubation period, 100 μl of media from the upper (apical) and lower (basolateral) chamber collected separately were placed in a 96-well plate and the concentration of FITC-Dextran was measured on a fluorescence multiplate reader (FLUOstar Omega, excitation, 486 nm; emission, 520 nm). Relative fluorescence was then calculated as a ratio between lower (basolateral) chamber fluorescence and total fluorescence. Sample readings were performed in triplicate.

### Adhesion of *E. coli* O157:H7 to HCT-8 cells

HCT-8 cells were grown on 24-wells plates until a confluent monolayer was achieved (approximately 6 x 10^5^ cells). Monolayers were treated at 37°C for 5 h with 1 x 10^8^ CFU/ml *E. coli* O157:H7 in minimal M9 medium (Sigma-Aldrich, USA supplemented with 2 mM MgSO_4_, 0.1 mM CaCl_2_ and 10 mM mucus-derived metabolites (Fucose, Galactose, N-Gal. NANA or NAG) or Glucose. To count CFU number, cells were washed five times with PBS to remove non-attached bacteria and lysed using 0.2% Triton-PBS solution for 30 min. Serial dilutions of these suspensions were spread on LB-agar coated Petri dishes and incubated at 37°C for 24 h for optical counting. Results were informed as total CFU quantified or relative to total HCT-8 cells (multiplicity of infection, MOI).

### Statistical analysis

Cytotoxicity curves were fitted using linear or logarithmic regression. Statistical significance for all assays was assessed using one-way ANOVA with Tukey’s or Bonferroni’s multiple comparison test or Student’s t-test. Data are shown as means ± SEM from five independent experiments performed in triplicate. Analysis was performed using Graphpad Prism v8.0.2 software. Statistical significance was set at *p < 0.05.

## Results

### Influence of mucus-derived metabolites on *E. coli* O157:H7 growth

To determine whether mucus-derived metabolites affected *E. coli* O157:H7 growth, bacterial cultures were grown alone or in the presence of HCT-8 cells.

**Figure 1:**
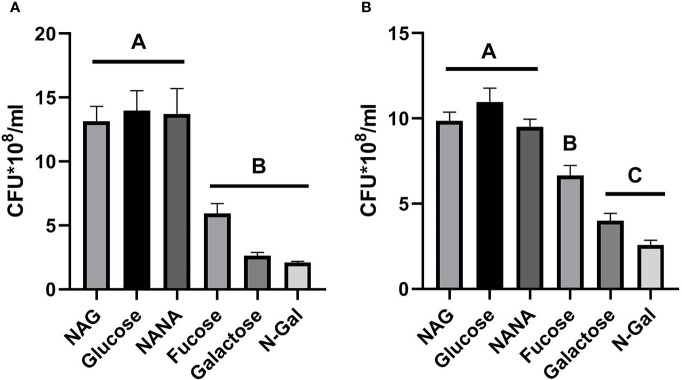
*E. coli* O157:H7 growth in the presence of mucus-derived sugars. *E. coli* O157:H7 cultures were grown in M9 medium +10 mM mucus-derived metabolites (Fucose, Galactose, N-Gal. NANA or NAG) or Glucose at 37°C for 5 h under agitation in absence of intestinal cells **(A)** or in the presence of HCT-8 cells monolayers grown on 24-well plates at 37°C in a humidified 5% CO_2_ incubator under static conditions **(B)**. CFU counting was performed on agar plates and is shown as CFU*10^8^. Data shown as mean ± SEM. Significant differences were found between groups with different letters (A, B and C) (p<0.05, n= 5).

**Figure 1A** shows that bacteria grown in the presence of NANA and NAG did not show significant differences in growth capability compared to cultures grown with Glucose, while bacteria grown with Galactose, N-Gal or Fucose as the only carbon source showed a significantly lower growth than the previous group, although bacterial growth with Fucose was slightly higher than the aforementioned ​​(p>0.05, NS, n= 5).

Regarding the growth of bacteria in the presence of HCT-8 cells (**Figure 1B**), a similar trend could be observed, although in this case, growth with Fucose was significantly greater than that achieved with Galactose and N-Gal and significantly lower than that observed with NAG, NANA and Glucose (p<0.05), suggesting that under co-culture conditions with intestinal cells, Fucose was more efficiently used as a carbon source by *E. coli* O157:H7 when compared to Galactose and N-Gal.

### Effect of mucus-derived metabolites on *E. coli* O157:H7 adhesion to HCT-8 cells

We analyzed the influence of mucus-derived metabolites on the ability of *E. coli* O157:H7 to adhere to HCT-8 cells.

**Figure 2:**
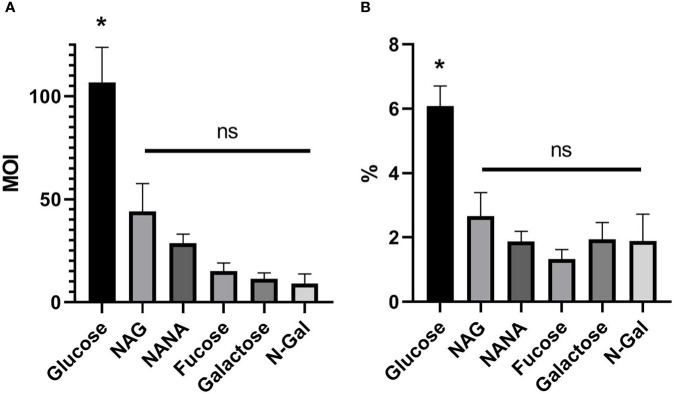
*E. coli* O157:H7 adhesion on HCT-8 cells in the presence of mucus-derived sugars. *E. coli* O157:H7 cultures were coincubated with HCT-8 monolayers in M9 medium + 10 mM mucus-derived metabolites (Fucose, Galactose, N-Gal. NANA or NAG) or Glucose at 37°C for 5 h at 37°C in a humidified 5% CO_2_ incubator. **(A)** Multiplicity of infection (MOI) for each treatment, calculated as number of adhered bacteria/number of total HCT-8 cells. **(B)** Percentage of adherent bacteria, expressed as adherent bacteria/total bacteria. Data shown as mean ± SEM (*p < 0.05, n = 5).

As it is shown in **Figure 2A**, MOI of *E. coli* O157:H7 incubated with Glucose was significantly higher than those incubated with any of the mucus-derived metabolites used (p<0.05). Likewise, the same difference was observed when analyzing the percentage of adhered bacteria, calculated as the total adhered bacteria with respect to the total bacteria present at the end of the incubation time for each treatment (**Figure 2B**). These results indicate that mucus-derived metabolites have an inhibitory effect on bacterial adhesion.

### Influence of mucus-derived metabolites on Stx2 production

Next, we assessed the effect of mucus-derived metabolites on the production of Stx2 by *E. coli* O157:H7.

**Figure 3:**
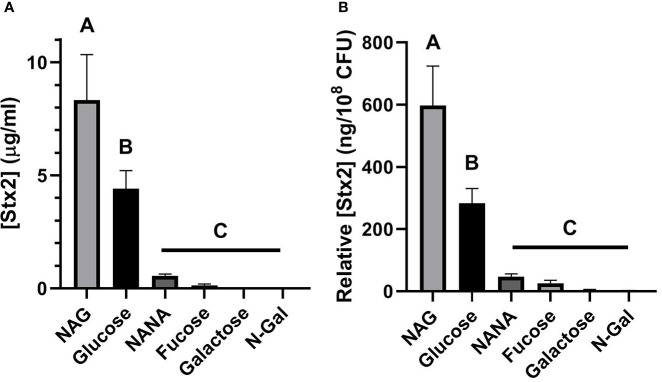
Production of Stx2 in the presence of mucus-derived sugars. E. *coli* O157:H7 cultures were grown in M9 +10 mM medium of mucus-derived metabolites (Fucose, Galactose, N-Gal. NANA or NAG) or Glucose at 37°C for 5 h under agitation. **(A)** [Stx2] in the filtered bacterial SN estimated from its cytotoxic activity in Vero cells. **(B)** [Stx2] relative to bacterial density, expressed as ng of Stx2 per 10^8^ CFU present after the incubation period. Data are shown as mean ± SEM. Significant differences were found between groups with different letters (A. B and C) (p < 0.05, n = 5).

As it is shown on **Figure 3A**, the filter-sterilized bacterial SN obtained from the cultures grown in the presence of NAG had maximum Stx2 yield, even when compared to those elicited by the bacterial SN grown with Glucose. The remaining metabolites elicited significantly lower Stx2 yields than NAG and Glucose (p<0.05, n=5). Identical results were obtained when Stx2 concentration was related to bacterial density, expressed as ng of Stx2 per 1 x 10^8^ CFU present after the incubation period (**Figure 3B**, p<0.05). These results suggest that production of Stx2 is strongly increased when bacteria uses NAG as the main carbon source, and decreases when any other metabolite is available.

### Effect of mucus-derived metabolites on Stx2 translocation across HCT-8 cell monolayers

We then assessed the effects of mucus-derived metabolites on Stx2 translocation through HCT-8 cell monolayers in the presence of *E. coli* O157:H7Δstx2 supplemented with 100 ng/ml Stx2 in order to make translocation independent of toxin production.

**Figure 4:**
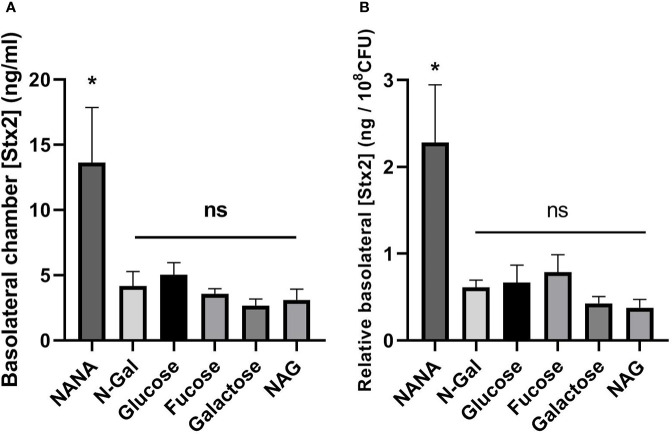
Translocation of Stx2 across the HCT-8 monolayer. E. *coli* O157:H7Δstx2 cultures + 100 ng/ml Stx2 supplemented with 10 mM Fucose, Galactose, N-Gal. NANA, NAG or Glucose were coincubated with HCT-8 monolayers grown on Milicell supports in M9 medium at 37°C for 5 h in a humidified 5% CO_2_ incubator. **(A)** Stx2 concentration (basolateral chamber) measured by Vero cell cytotoxicity assays. **(B)** Translocated Stx2 concentration (basolateral chamber) relative to the bacterial density found in the apical chamber after incubation, expressed as ng of Stx2 per 10^8^ CFU. Data shown as mean ± SEM. *p < 0.05, n = 5.

Stx2 translocation was significantly higher when bacteria were incubated with NANA as the sole carbon source compared to the rest of the treatments, including Glucose (**Figure 4A**, p<0.05). This difference was also significant when Stx2 passage was related to the total bacterial load after incubation (**Figure 4B**, expressed as translocated [Stx2] per 10^8^ CFU, p<0.05). These results indicate that the presence of NANA as a sole carbon source strongly stimulates the translocation of Stx2 through the epithelium.

**Figure 5:**
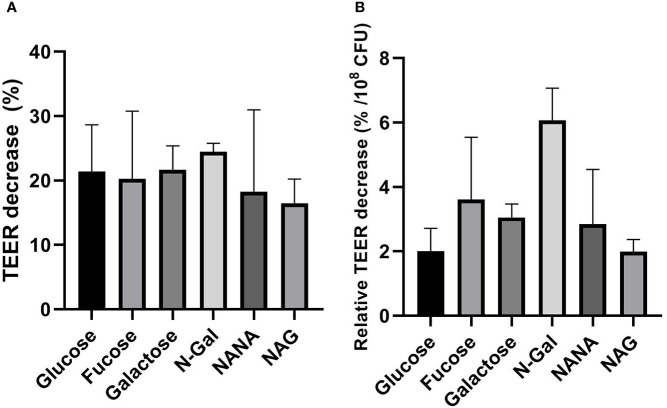
Influence of mucus-derived metabolites on transepithelial resistance (TEER) decrease of HCT-8 monolayers caused by *E. coli* O157:H7. *E. coli* O157:H7Δstx2 + 100 ng/ml Stx2 cultures were coincubated with HCT-8 monolayers grown on Milicell supports in M9 medium supplemented with 10 mM mucus-derived metabolites (Fucose, Galactose, N-Gal. NANA or NAG) or Glucose at 37°C for 5 h in a humidified 5% CO_2_ incubator. **(A)** Percentage decrease of TEER after treatments. **(B)** Percentage TEER decrease after treatments relative to bacterial density measured in the apical chamber after treatments, expressed as percentage decrease per 10^8^ CFU. Data shown as mean ± SEM. No significant differences were found between treatments (p > 0.05, n = 5).

To analyze the effect of the treatments on the paracellular permeability of the epithelial monolayer, TEER and Dextran-FITC passage were measured before and after each treatment. Under the experimental conditions described, no significant differences were found in the TEER decrease after treatment, both in absolute value (**Figure 5A**) and relative to the bacterial load after incubation (**Figure 5B**). These results show that the carbon source used has no influence over the loss of integrity of the epithelial barrier.

**Figure 6:**
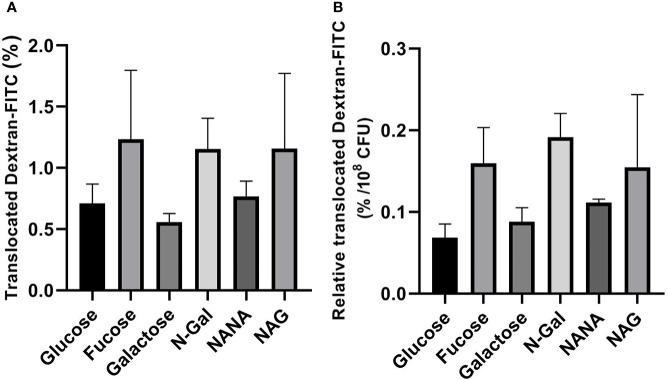
Dextran-FITC translocation across HCT-8 monolayers. E. *coli* O157:H7Δstx2 + 100 ng/ml Stx2 cultures were coincubated with HCT-8 monolayers grown on Milicell supports with M9 medium supplemented with 10 mM mucus-derived metabolites (Fucose, Galactose, N-Gal. NANA or NAG) or Glucose at 37°C for 5 h in a humidified 5% CO_2_ incubator. **(A)** Percentage translocated Dextran-FITC (basal chamber) calculated as fluorescence measured in the basal chamber/total fluorescence*100. **(B)** Percentage translocated Dextran-FITC (basal chamber) relative to bacterial density found in the apical chamber after treatments, expressed as percentage of translocated Dextran-FITC per 10^8^ CFU. Data shown as mean ± SEM. No significant differences were found between treatments (p > 0.05, n = 5).

In the same direction, no significant differences were found in the passage of Dextran-FITC (**Figures 6A, B** **)** regardless of the carbon source used. Altogether, these results indicate that differences found in Stx2 passage cannot be attributed to an increase in the paracellular route.

## Discussion

In this study we demonstrated that the main metabolites derived from the degradation of intestinal mucus affect different variables associated with the pathogenicity of *E. coli* O157:H7. First, the growth capacity of *E. coli* O157:H7 in M9 medium was evaluated using either 10 mM of the mucus-derived metabolites (Fucose, Galactose, N-Gal, NANA, NAG) or Glucose, a sugar not found at the level of the colon (Holst et al., 2016), as the sole carbon source. Although *E. coli* O157:H7 was able to grow in the presence of all sugars, bacteria proliferated significantly more on Glucose, NANA or NAG than on Galactose, N-Gal and Fucose, although the latter tended to be located on intermediate values ​​(**Figure 1A**). These results agree with those reported in the literature for a similar model of monosaccharide utilization in minimal medium, where NAG and NANA were the most efficiently used carbohydrates (Bertin et al., 2013). When evaluating growth under co-culture conditions with human colonic epithelial cells (HCT-8), the difference in growth observed in the presence of Fucose became statistically significant compared to the rest of sugars (**Figure 1B**).

The relative growth advantage of Fucose over N-Gal or Galactose might be explained by the repression of the LEE pathogenicity island induced by this sugar, which confers an adaptive advantage over growth with other carbon sources, as this system is energetically very expensive (Pacheco et al., 2012). This difference becomes particularly noticeable under co-culture conditions with the colonic epithelium, as this is a strong stimulator of LEE (Bansal et al., 2013).

Furthermore, adhesion capacity of *E. coli* O157:H7 to the colonic epithelium was analyzed, as it is one of the most important virulence factors associated with bacterial pathogenicity. The adhesion capacity of the bacterial cultures was measured both absolutely (reported as adhered bacteria per epithelial cell, MOI, **Figure 2A**) and relatively to the total bacterial growth at the end of the co-culture (**Figure 2B**). Our results showed that all the mucus-derived metabolites inhibited bacterial adhesion compared to Glucose. Previous studies have shown that the adhesion of *E. coli* in the presence of NAG or NANA is decreased, either by nonspecific mechanisms dependent on fimbriae (Plumbridge and Vimr, 1999; Sohanpal et al., 2004; Barnhart et al., 2006), or by specific mechanisms dependent on negative regulation of LEE (Le Bihan et al., 2017), as was also described for Fucose (Pacheco et al., 2012).

In this study we demonstrate that the presence of Galactose or N-Gal is also capable of repressing the adhesive capacity of *E. coli* O157:H7 to the colonic epithelium. In the case of Galactose, we hypothesize that this is possibly due to the fact that the NagC regulator, responsible for the repression of LEE in the presence of NAG, has similar functionality in the metabolic pathway of this sugar (El Qaidi et al., 2009). In the case of N-Gal, it is known that part of its metabolism is also related to the NAG metabolic pathway (Hu et al., 2013)and that deletions in this pathway cause colonization defects in cattle (Snider et al., 2009), so it is possible that this regulation exerts a similar effect in the inhibition of adhesion to human colonic epithelial cells.

Also, recent studies reported that the expression of EspB, a virulence factor associated with adhesion and encoded in the LEE, is repressed using Galactose, Fucose and Glucose and increases with NAG, NANA and N-Gal as the only carbon source (Carlson-Banning and Sperandio, 2016). In contrast, in adhesion assays of *E. coli* O157:H7 to HeLa cells, it was reported that LEE is inhibited when NAG and NANA are used as carbon source, but no differences were found with Galactose and N-gal (Le Bihan et al., 2017). Even when there is a disparity of information regarding how the different carbon sources modulate bacterial growth and adhesion, it is possible to conclude that mucus-derived sugars are essential to modulate EHEC colonization and their study is necessary to find new strategies that can avoid it (Woodward et al., 2019).

Although numerous studies have evaluated the role of Stx2 in its relation with the commensal cattle or human microbiota in the gut (Lee et al., 2021), the effect of mucus-derived sugars on Stx2 production has not been explored so far. In this study, we found that both the net (**Figure 3A**) and relative production of Stx2 (**Figure 3B**) were increased when the sole carbon source used was NAG. In contrast, a decrease in Stx2 production was observed when any other mucus-derived metabolite was used, compared to Glucose.

A widely accepted hypothesis is that *E. coli* O157:H7 modifies its virulent profile in response to environmental sensing. An environment rich in sugars derived from mucus would indicate the presence of commensal microbiota with mucolytic activity (Tailford et al., 2015). This environment would be incompatible with proximity to the colonic epithelium, which is generally sterile, so genes associated with virulence, such as the LEE, would be repressed (Kamada et al., 2012; Pacheco et al., 2012; Cameron and Sperandio, 2015; Le Bihan et al., 2017). In this sense, we hypothesize that the production of Stx2 could be controlled by similar regulatory mechanisms. Furthermore, this environment would not be associated with the proinflammatory conditions required to trigger the production of Stx2 and its associated phage (Exeni et al., 2018). We also hypothesize that the increased expression of Stx2, and consequently of its phage, in the presence of NAG may have a dual explanation. First, the colonic epithelium presents high levels of O-NAG-Acylations on the cell surface of enterocytes, which could be sensed by *E. coli* O157:H7 and represent an indicator of proximity to the epithelium and, therefore, of a proinflammatory environment. Furthermore, Stx2 is known to induce overexpression of O-NAG-Acylations in host cell proteins, conferring a boost to this signal (Lee et al., 2022). Another possible explanation for the increase in Stx2 in the presence of NAG may be associated to the evolutionary origin of the incorporation of the Stx2-associated phage in *E. coli*, which is accepted to be related to the toxicity that it produces on its natural predators and competitors, such as protists and fungi (Steinberg and Levin, 2007; Koudelka et al., 2018).Taking into account that chitin is the main component of the fungal cell wall (Min et al., 2020), that this molecule is a polymer of NAG easily catabolizable to its constituent monosaccharides (Hui et al., 2020), and that NAG is already used as a modulator of virulence by *E. coli* O157:H7 (Sohanpal et al., 2004; Konopka, 2012; Le Bihan et al., 2017; Sicard et al., 2018) we hypothesize that a high amount of this sugar could be used by *E. coli* O157:H7 as a signal indicating an unfavorable environment related to a prevalence of fungi in the intestinal environment.

Furthermore, we evaluated the ability of mucus-derived metabolites to modulate the effects of *E. coli* O157:H7 on Stx2 translocation across a monolayer of human intestinal epithelial cells (HCT-8). For this purpose, we coincubated cultures of *E. coli* O157:H7Δstx2 with monolayers of HCT-8 cells grown on Milicell permeable supports in M9 minimal medium supplemented with Stx2 and the sugars derived from mucus or Glucose as sole carbon sources. Determinations of Stx2 translocated to the basolateral chamber revealed a significant increase in translocation when bacteria were incubated with NANA when compared to both Glucose and the rest of the metabolites (**Figure 4**). As we previously described (Garimano et al., 2019), translocation of Stx2 can occur through the paracellular and/or transcellular pathways. To assess which of these pathways was stimulated, the passage of Dextran-FITC, associated with paracellular permeability of the monolayer, and TEER, as an indicator of the integrity of the epithelial barrier tight junctions, were simultaneously measured, pre- and post- treatments. No significant differences were observed in the passage of Dextran-FITC (**Figure 5**) or in decrease in TEER (**Figure 6**) between treatments, indicating that the paracellular translocation pathway was not increased. These results indicate that the stimulation of the passage of Stx2 observed in the presence of NANA must have exclusively occurred through the transcellular pathway, although the precise mechanisms underlying this stimulation are still not clarified.

The relationship of NANA on both the metabolism and virulence of EHEC has been previously explored. First, NANA is known to be an important nutritional source for EHEC in both cattle (Segura et al., 2018) and mice (Fabich et al., 2008), and alternative O-acetylated sources of this sugar can also be metabolized by esterases encoded both genomically and in the prophage associated with Stx2 (Haines-Menges et al., 2015; Rangel et al., 2016; Saile et al., 2018). The sensing and metabolism of NANA was previously implicated in different processes related to virulence, finding that it inhibits the specific and non-specific adhesion of EHEC to epithelial cells (Le Bihan et al., 2017) and, at the same time, increases the secretion of virulence factors associated with T3SS, as is EspB (Carlson-Banning and Sperandio, 2016). Some authors have pointed out that an increase in the transcellular translocation of Stx2 is produced independently of bacterial adhesion (Tran et al., 2018), implicating other virulence factors secreted into the medium by the bacteria, such as EspP in the process (In et al., 2013). We therefore hypothesize that the increase in Stx2 transcytosis stimulated by NANA could be related to an increase in the virulence factors secreted by *E. coli* O157:H7 to the media, and independently of bacterial adhesion.

Alternatively, it is known that the addition of NANA-like sugars, such as Neu5Gc, to the culture medium promotes the synthesis of specific toxin receptors of the AB5 family in human colonic epithelium (Byres et al., 2008). Although the Stx2-specific glycolipid receptor, Gb3, does not contain NANA in its structure (Zhang et al., 2012), it shares the same metabolic precursor (LacCer) with the group of gangliosides that do present this sugar in their glycosidic portion (Yager and Konan, 2019). As it has been shown that imbalances in this metabolic pathway can affect the levels of Gb3 synthesized (Kroes et al., 2010), we hypothesize that the addition of NANA as a carbon source could modulate the Gb3 expression profile of HCT-8 cells, making them more susceptible to the incorporation and subsequent translocation of Stx2, but further studies are needed in this direction.

In summary, our results indicate that the production and translocation of Stx2 through EHEC-infected human colonic epithelium can be modulated by mucus derivatives independently of bacterial proliferation and adhesion. In this experimental context, we generated new knowledge about the host-microbiota-pathogen interaction and its importance regarding the incidence and severity of HUS in patients infected with EHEC.

## Conclusion

This work provides information about the influence of the mucolytic commensal microbiota, through the sugars released by its action, on the pathogenicity of *E. coli* O157:H7, by affecting its colonization capacity, in addition to the production and translocation of Stx2 towards the systemic circulation. These studies collaborate with the identification of favorable intestinal environments for the development of HUS that can explain the diverse development of the disease between different patients and shed light towards the prevention of severe outcomes.

## Data availability statement

The raw data supporting the conclusions of this article will be made available by the authors, without undue reservation.

## Author contributions

All authors listed have made a substantial, direct and intellectual contribution to the work, and approved it for publication.

## Funding

This study was supported by the National Scientific and Technical Research Council (CONICET PUE-2017-0041.

## Conflict of interest

The authors declare that the research was conducted in the absence of any commercial or financial relationships that could be construed as a potential conflict of interest.

## Publisher’s note

All claims expressed in this article are solely those of the authors and do not necessarily represent those of their affiliated organizations, or those of the publisher, the editors and the reviewers. Any product that may be evaluated in this article, or claim that may be made by its manufacturer, is not guaranteed or endorsed by the publisher.
